# Changes in cognitive-motor interference during rehabilitation of cane walking in patients with subacute stroke: A pilot study

**DOI:** 10.1371/journal.pone.0274425

**Published:** 2022-10-06

**Authors:** Hsiu-I Chen, Shu-Yi Fu, Ting-Wei Liu, Ya-Wen Hsieh, Hui-Ya Chen

**Affiliations:** 1 Department of Physical Therapy, Hung Kuang University, Taichung, Taiwan; 2 Physical Therapy Room, Chung Shan Medical University Hospital, Taichung, Taiwan; 3 Bentang Cheng Ching Hospital Nursing Home, Taichung, Taiwan; 4 Outstanding Physiotherapy Clinic, Taichung, Taiwan; 5 Department of Physical Therapy, Chung Shan Medical University, Taichung, Taiwan; Universidade Federal do Rio Grande do Sul, BRAZIL

## Abstract

No previous research has examined cognitive-motor interference (CMI) repeatedly in patients with subacute stroke. This pilot study aimed to report on the changes over time in CMI in patients with stroke who have recently learned to walk with a cane. The assessment started as soon as the participants could walk independently with a quad cane, and was repeated up to six sessions as long as the cane was still used. The dual-tasking paradigm required participants to walk and perform continuous subtractions by 3s. Data were analyzed for 9 participants 33–127 days post-stroke. All 9 participants showed CMI in walking velocity at baseline and 8 of these showed improvement over time (Z = -2.547; p = 0.011). The improvement in CMI was associated with baseline dual-tasking performance (ρ = 0.600; p = 0.044), motor control ability (ρ = -0.695; p = 0.019), walking velocity (ρ = -0.767; p = 0.008), and functional mobility (ρ = 0.817; p = 0.004). All participants showed decrements in both tasks (mutual interference) at baseline, 1 evolved to decrements in walking velocity (cognitive-related motor interference), and 3 finally evolved to decrements in cognitive performance but increments in walking velocity (motor-priority tradeoff). In conclusion, during rehabilitation with cane walking in patients with subacute stroke, the dual-tasking paradigm revealed CMI and its improvements in the majority of participants. Greater improvement in CMI was moderately to strongly associated with worse baseline performance of many variables. The evolution of the CMI pattern over time provides novel information relevant to neurological recovery.

## Introduction

In contrast to the traditional view that human gait relies only on motor and sensory functions, research has shown that human gait also relies on executive functions such as attention and working memory [1,2]. The attentional demand of human gait could be manifested by the dual-tasking paradigm, which mimics real-life challenges by asking participants to concurrently execute a motor task and a cognitive task [1,2]. Cognitive-motor interference (CMI), defined as a deterioration in performance while dual-tasking, is typically interpreted as a result of competing demands for the attentional resources required for both tasks and primarily depends on one’s ability to properly allocate attention between the two tasks [3]. A higher CMI implies greater demand of attentional resources, whereas a decline of CMI indicates the trend toward automaticity [4].

Many studies have reported CMI in the gait of patients with stroke [4–13], which may manifest in one or both tasks. However, most relevant studies only recruited patients with chronic stroke, with an average time of over 6 months since the onset of stroke [4,5,7–10,12,13]. In addition, only one study by Cockburn et al. [6] investigated CMI repeatedly in stroke gait. They tested the dual-tasking paradigm in 10 patients (average time since stroke onset of 5.7 months, range 1–10 months), once during inpatient rehabilitation and a retest 1 to 9 months later. At the initial assessment, four participants were considered to be at a relatively good stage of clinical recovery, but still showed CMI in gait performance. Over time, CMI reduced in most participants while they were relearning to walk: 7 out of 10 showed CMI reduction in terms of gait performance, whereas 3 out of 10 showed CMI reduction in terms of cognitive performance. However, inferential statistics were not performed, and the presence, type, or change of walking aids used by the participants was not described.

Apart from the above quantitative research, in a review paper on CMI of stroke gait, Plummer et al. [14] categorized CMI into nine possible patterns. They found that patients with stroke are more likely to show decrements in gait performance alone (cognitive-related motor interference) or in both gait and cognitive performance (mutual interference), and they are less likely to exhibit impaired cognitive performance alone (motor-related cognitive interference). In other words, gait performance is more likely to be influenced than cognitive performance during dual-tasking.

In rehabilitation, most stroke survivors lose their ability to walk and need intensive practice with a walking aid, such as a quad cane, in order to achieve an efficient and safe walking performance. The use of walking aids is a learning process [15] that requires attentional demand [16–18] before a certain level of automaticity is regained. How CMI changes in patients in the early stages of relearning to walk with a cane in the subacute stage, including the extent of CMI and the individual (not group average) CMI pattern, is key to revealing an individual’s neurological recovery. Plummer et al. [14] proposed an interesting hypothesis that the CMI pattern may evolve with stroke chronicity. For example, in the inpatient rehabilitation phase, dual-tasking mainly results in mutual interference [5,6], and the pattern of cognitive-related motor interference is evident 7–8 months post stroke [7], whereas no interference is reported after 2 years following the stroke [13].

The above literatures suggest that the extent and pattern of CMI appear to be good indicators of functional recovery during gait rehabilitation in patients with stroke; however, no previous study has examined this repeatedly in patients with subacute stroke. The purpose of this pilot study was to report the quantity and quality changes of individual CMI during gait rehabilitation in patients with subacute stroke. The assessment started immediately after participants could walk with a quad cane without physical assistance from others and was repeated as many sessions as possible for a total of six sessions in 3 months. There were three specific aims: (1) to test the feasibility of the dual-tasking paradigm in the subacute stroke stage when patients have just learnt to walk with a quad cane, (2) to report changes over time in an individual’s extent and pattern of CMI, and (3) to examine the relationship between changes in the extent of CMI and other factors, including age, stroke severity, stroke onset period, and functional mobility.

## Materials and methods

### Design

The assessment started immediately after participants could walk with a quad cane without physical assistance from others and was repeated as many sessions as possible for a total of six sessions in 3 months. As long as the cane was still used, the dual-tasking paradigm, which required participants to walk and perform continuous subtractions by 3s, was repeatedly conducted in each session. In order to show the learning effect of cane walking, the number of days since first cane use was calculated as the time line of each testing session.

### Participants

The sample size was calculated by G*Power 3.1 [19]. The baseline CMI value (17.33±4.9%) was based on three data points of patients one- to two-month post-stroke selected from Cockburn et al. [6], and the second CMI value (11.0±4.4%) was drawn from Dennis et al. [13] in which the same serial subtractions of 3s task was used in chronic stroke patients. With alpha set at 0.05 and power set at 0.95, the minimum sample size needed was 8.

The participants were a cohort admitted to the rehabilitation wards during July 2016 –May 2019. The exclusion criteria were: (1) hemi-neglect; (2) pusher syndrome; (3) prior experience of walking with walking aids; (4) a major neurologic or musculoskeletal diagnosis other than stroke; and (5) unstable medical conditions. The inclusion criteria were: (1) first-ever stroke onset on only one side of the brain; (2) ability to follow simple instructions and solve simple mathematical tasks; and (3) ability to walk on a levelled surface with a quad cane but without personal help for 5.56 meters (supervision or a contact guard was allowed), within 60 days since the first experience of cane use. All participants signed an informed consent form, and the study was approved by the Institutional Review Board of Chung Shan Medical University Hospital. The authors had access to information that could identify individual participants during and after data collection.

### Procedure

If the first two inclusion criteria were met, potential participants were frequently monitored to ensure that they met the last criterion. The baseline session was scheduled as soon as the physical therapists confirmed that the patient could walk on a levelled surface with a quad cane, but without personal help for 5.56 meters. At baseline, we obtained data on cognitive function as assessed by mini-cog [20], functional mobility as assessed by the Timed Up and Go (TUG) test with the prescribed quad cane [21], stroke severity, motor control ability as assessed by the lower extremity part of the Fugl-Meyer Assessment (F-M LE) [22], and function of daily living activities as assessed by the Barthel index (BI) [23].

**Fig 1:**
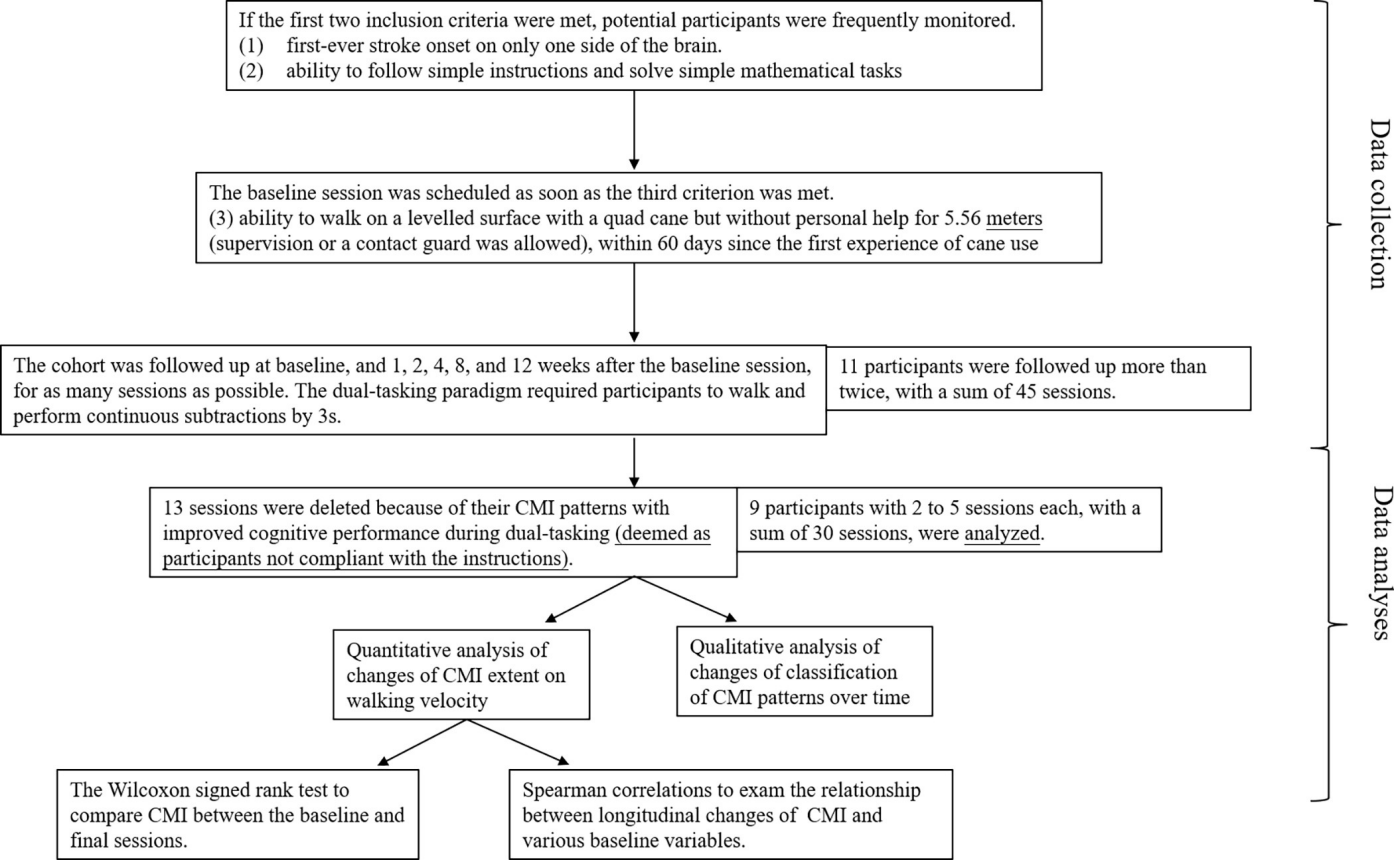
Flowchart of data collection and data analyses. CMI is the abbreviation of cognitive-motor interference.

The cohort was followed up as many sessions as possible for a total of six sessions, at baseline, and at 1, 2, 4, 8, and 12 weeks after the baseline session (Fig 1). As long as the patient was still using the prescribed quad cane as a walking aid and was still willing to participate, regardless of whether they had been transferred to another hospital for rehabilitation or had already been discharged from hospital, we collected data from as many sessions as possible. Based on the law of practice [24], we expected larger changes in earlier learning phases; therefore shorter intervals were scheduled for the earlier phases.

In each session, the dual-tasking paradigm required participants to walk with the prescribed quad cane and to solve continuous subtraction tasks simultaneously. Gait analysis was performed using an electronic walkway (GAITRite, CIR systems, Inc., Franklin, NJ, USA) with sensor pads placed over a length of 4.26 meters. For acceleration and deceleration, the walkway was extended by two yoga mats at both ends with matched height and similar softness with the GAITRite mat, thus creating a walking path of 5.56 meters.

The mathematical task was chosen as the cognitive task. Among the various cognitive tasks conducted in parallel with gait, mathematical tasks have been reported to be uniquely influenced by focused attention [25], to disturb gait performance the most in neurological patients [26], result in the highest cognitive cost in patients with stroke [27], and to best predict elderly falls [28]. There were two levels of subtraction tasks, a low-demand task involving deductions of 1s and a high-demand task involving serial deductions of 3s from a random number between 60 and 100. The low-demand subtraction task served as the control condition because, according to a review paper by Fraizer and Mitra [29], a pure single-task baseline condition is problematic with no control over what participants think about.

During the measurements, the participants walked on a walkway at a comfortable pace while wearing their usual low-heel shoes and ankle-foot-orthoses, if applicable. Participants were asked to wear the same shoes throughout the repetitive tests. To measure steady state walking, the participants started walking from one end of the walkway toward a chair positioned at the other end of the walkway. Because the participants had just learnt to walk again and were easily fatigued, no practice trials were conducted, and only two trials for each condition were conducted. The order of trials was fixed in a reverse counterbalanced order: (1) walking with a low-demand subtraction task, (2) sitting with a low-demand subtraction task for 20 seconds, (3) walking with a high-demand subtraction task, (4) sitting with a high-demand subtraction task for 20 seconds, (5) sitting with a high-demand subtraction task for 20 seconds, (6) walking with a high-demand subtraction task, (7) sitting with a low-demand subtraction task for 20 seconds, and (8) walking with a low-demand subtraction task. A researcher walked alongside participants to ensure their safety, to provide the number used for the continuous subtraction tasks when the participants approached the 4.26-meter markers, and to record the walking duration of the middle 4.26-meter walk using a stopwatch. Participants were asked to orally pronounce their answers continuously and to prioritize both tasks.

### Data analysis

Gait and cognitive performance in the middle 4.26-meter walk were analyzed. Al-Yahya et al. [26] reported in a meta-analysis that walking velocity is the gait parameter most influenced by a dual-tasking paradigm and thus was chosen to represent gait performance. In cases where the walking velocity could not be automatically outputted by GAITRite due to reasons such as a dragging gait or overlapped strides, data from manual timing with a stopwatch were used. The number of mathematically correct answers per second was recorded as the cognitive performance. For both gait and cognitive performance, larger values indicated better performance.

In our quantitative analysis, the CMI of the walking velocity was calculated as ((low-demand task—high-demand task)/low-demand task) × 100% [6,30]. For example, if the walking velocity of low-demand task was 0.124 m/s and that of high-demand task was 0.091 m/s, CMI would be 26.61%. A positive value indicated a deterioration in walking velocity due to dual-tasking. Changes in the extent of CMI were calculated individually by subtracting the CMI of the final session from the CMI of the baseline session. For example, if the CMI at baseline was 26.61% and that at final session was -0.32%, the changes in CMI would be 26.93%. A positive value indicated a trend of improvement, and larger values indicated greater improvement. Data were analyzed using the SPSS statistical package (PASW Statistics 14.0, SPSS Inc., Chicago, USA). The Wilcoxon signed rank test was used to examine changes in the extent of CMI between the baseline and final sessions, with the significance level set at 0.05.

In our qualitative analysis, the CMI pattern in each session of a participant was categorized into six patterns: decrements in both gait and cognitive performance (mutual interference), in gait performance only (cognitive-related motor interference), in cognitive performance only (motor-related cognitive interference), or in no task (no interference), and improvements in gait performance with no change in cognitive performance (motor facilitation) or with worsened cognitive performance (motor-priority tradeoff). In a review article, Plummer et al. [14] categorized CMI into nine possible patterns, but found that three CMI patterns with improved cognitive performance during dual-tasking rarely occurred in the literature. These three CMI patterns may indicate that participants did not comply with the instructions to prioritize both tasks. Thus, they were not considered in this study.

In order to examine the relationship between the changes over time in the extent of CMI and various baseline variables, including age, time since stroke onset, time since first cane use, baseline CMI, walking velocity during low-demand dual-tasking, F-M LE, BI, and TUG, Spearman correlations were performed with the significance level set at 0.05. Correlation coefficients of less than 0.25, between 0.25 and 0.50, between 0.50 and 0.75, and greater than 0.75 indicated little or no relationship, a fair relationship, a moderate-to-good relationship, and a good-to-excellent relationship, respectively [31]. The time since stroke onset was defined as the time of date of stroke onset to the testing date. The time since first cane use was defined as the time from the date of first experience of cane use to the testing date.

## Results

### Feasibility of the dual-tasking paradigm of cane walking in patients with subacute stroke

There were 11 participants who had been tested more than 2 sessions, with a sum of 45 sessions. Out of the 45 sessions 13 sessions, distributed in 8 participants, showed better cognitive performance during cane walking than in sitting, which were deemed as participants not compliant with the instructions and were eliminated from further analyses.

After the 13 sessions were eliminated, there were 9 participants who still had more than 2 sessions. These 30 sessions, with 2 to 5 sessions per participant, were used in the analyses. Among the 30 sessions, the walking velocity in 6 sessions could not be automatically outputted by GAITRite in either the low- or high-demand condition, and therefore, we used the time durations obtained using a stopwatch in these 6 sessions.

Table 1 shows the basic demographics and characteristics of 9 participants tested at baseline. There were three women and six men, three patients with ischemic and six hemorrhagic strokes, and four left- and five right-sided hemiplegics. The median (range) values for age, body height, body weight, time since stroke onset, and time since first cane use were 57.4 (49.8–82.3) years, 164.0 (148.0–179.0) cm, 69.0 (42.0–89.0) kg, 60 (33–127) days, and 20 (6–51) days, respectively. The longest period after stroke onset at the final session was 173 days, which was still within 6 months of stroke onset. The median (range) values for education, mini-cog, and correct mathematical answer per second of continuous subtraction by 3s when seated were 11 (2–17) years, 5 (4–5), and 0.19 (0.13–0.55), respectively. The median (range) value for comfortable walking velocity during low-demand dual-tasking was 0.12 (0.05–0.39) m/s. The median (range) values for TUG, F-M LE, and BI were 85.9 (29.1–173.2) s, 22 (14–33), and 50 (35–90), respectively.

**Table 1:** Basic demographic and characteristics at baseline in each participant.

Participant ID	Sex	Age (year)	Body height (cm)	Body weight (kg)	Etiology	Hemiplegic side	Time since onset (day)	Time since first cane use (day)	Education (year)	mini-cog (0–5)	Correct mathematical answer (/s)^a^	Comfortable walking velocity (m/s)^b^	TUG (s)	F-M LE (0–34)	BI (0–100)
1	Woman	49.8	154	89.0	Hemorrhagic	Right	54	6	6	4	0.43	0.12	90.1	22	50
2	Man	64.9	170	72.0	Ischemic	Right	68	8	12	5	0.38	0.08	101.9	24	35
3	Man	53.1	164	66.0	Hemorrhagic	Right	45	20	11	5	0.13	0.12	85.9	14	55
4	Man	72.2	171	70.0	Ischemic	Right	115	30	2	5	0.18	0.05	173.2	16	50
5	Man	82.3	160	50.2	Ischemic	Left	33	10	6	4	0.13	0.05	141.5	19	40
6	Woman	62.6	148	52.4	Hemorrhagic	Right	127	38	17	5	0.55	0.14	55.7	17	60
7	Man	56.6	179	88.0	Hemorrhagic	Left	49	15	17	5	0.13	0.24	43.2	33	50
8	Man	50.4	168	69.0	Hemorrhagic	Left	122	51	12	5	0.39	0.39	29.1	31	90
9	Woman	57.4	150	42.0	Hemorrhagic	Left	60	48	6	4	0.19	0.23	33.9	31	90

Abbreviations: TUG, Timed Up and Go test; F-M LE, lower extremity subscores of the Fugl-Meyer assessment; BI, Barthel index.

^a^Correct mathematical answer per second of continuous subtraction by 3s when seated.

^b^Comfortable walking velocity during continuous subtraction by 1s.

### Changes in the extent and pattern of CMI

**Fig 2:**
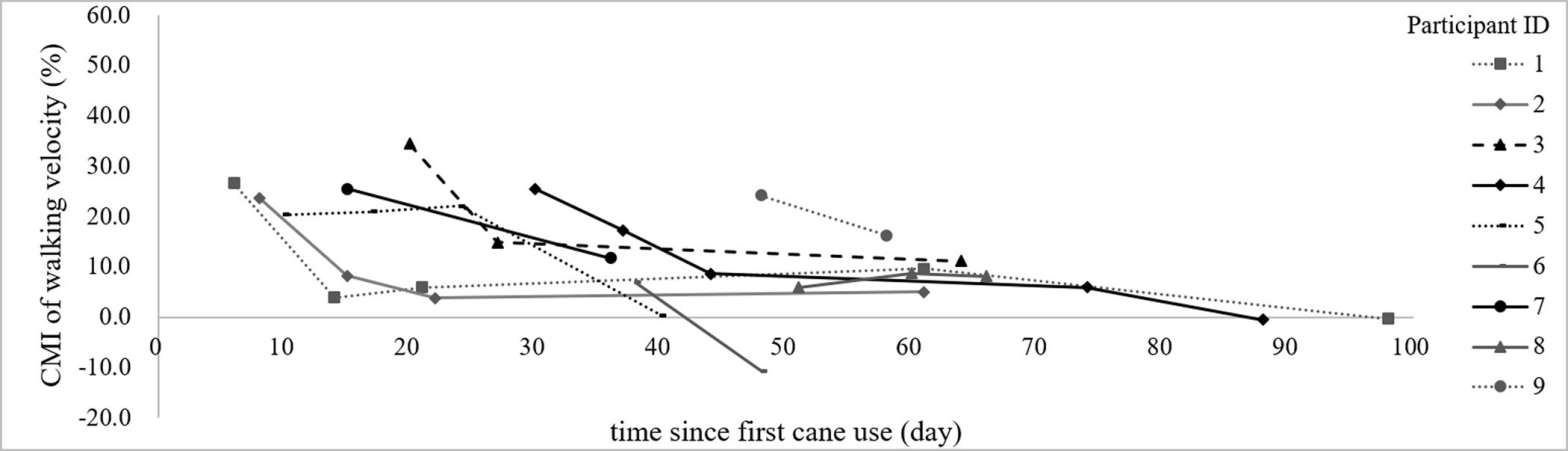
Changes in CMI as a function of number of days since first cane use in each participant. The x axis indicates number of days since first cane use, and each data dot represents one testing session. The y axis is the extent of CMI, with a positive value indicates a deterioration in walking velocity due to dual-tasking. The Wilcoxon signed rank test, performed between the baseline and final sessions, confirmed a significant improvement over time (Z = -2.547; p = 0.011).

Fig 2 and Table 2 reveal the changes over time in the extent of CMI in walking velocity. All 9 participants had positive CMI at the baseline session, which indicated a deterioration in walking velocity due to dual-tasking. Furthermore, 8 of 9 participants showed positive changes in CMI, which indicated improvement in CMI from the baseline to their final session. In 4 of 6 participants with more than 3 testing sessions, a trend towards larger changes in the earlier learning phases was observed. The Wilcoxon signed rank test confirmed a significant improvement between the baseline and final sessions (Z = -2.547; n = 9; p = 0.011). The median (range) value of daily changes in the extent of CMI was 0.53 (-0.15–1.03)%.

**Table 2:** CMI of walking velocity in each participant.

Participant ID	CMI at baseline session (%)^a^	CMI at final session (%)^a^	Changes in CMI (%)^b^	Daily changes in CMI^c^
1	26.61	-0.32	26.93	0.29
2	23.75	5.05	18.70	0.35
3	34.45	11.15	23.30	0.53
4	25.49	-0.50	25.99	0.45
5	20.37	0.36	20.01	0.67
6	6.94	-10.60	17.54	1.03
7	25.51	11.80	13.71	0.65
8	5.91	8.19	-2.28	-0.15
9	24.25	16.30	7.95	0.80

Abbreviation: CMI, cognitive-motor interference.

^a^CMI test at baseline or in the final session, calculated as ((low-demand task—high-demand task)/low-demand task) × 100%. The low- and high-demand tasks involved subtracting by 1s and 3s, respectively. A positive value indicated deterioration in walking velocity due to dual-tasking.

^b^Change in CMI, calculated as (CMI at baseline session—CMI at final session); a positive value indicated a trend towards improvement, and the larger the value, the greater the improvement.

^c^Daily change in CMI calculated as (change in CMI/the time interval between the final and baseline sessions).

Table 3 demonstrates the changes over time in the CMI pattern. Among the 6 possible CMI patterns, only 3 were evident in our study. All participants showed mutual interference at baseline. As time progressed, 1 participant showed cognitive-related motor interference, and 3 participants showed a motor-priority tradeoff in the final session.

**Table 3:** Changes in the CMI pattern as a function of day since first cane use in each participant. Each number indicates the days since first cane use, for each testing session.

Participant ID	CMI pattern						
	Mutual interference^a^					Cognitive-related motor interference^b^	Motor-priority tradeoff^c^
1	6	14	21			61	98
2	8	15	22	61			
3	20	27	64				
4	30	37	44	74			88
5	10	17	24	40			
6	38						48
7	15	36					
8	51	60	66				
9	48	58					

Abbreviation: CMI, cognitive-motor interference.

^a^Decrement in both gait and cognitive performance due to dual-tasking.

^b^Decrement in gait performance with no change in cognitive performance due to dual-tasking.

^c^Improvement in gait performance with worsened cognitive performance due to dual-tasking.

### Relationship between changes in extent of CMI and other factors

**Fig 3:**
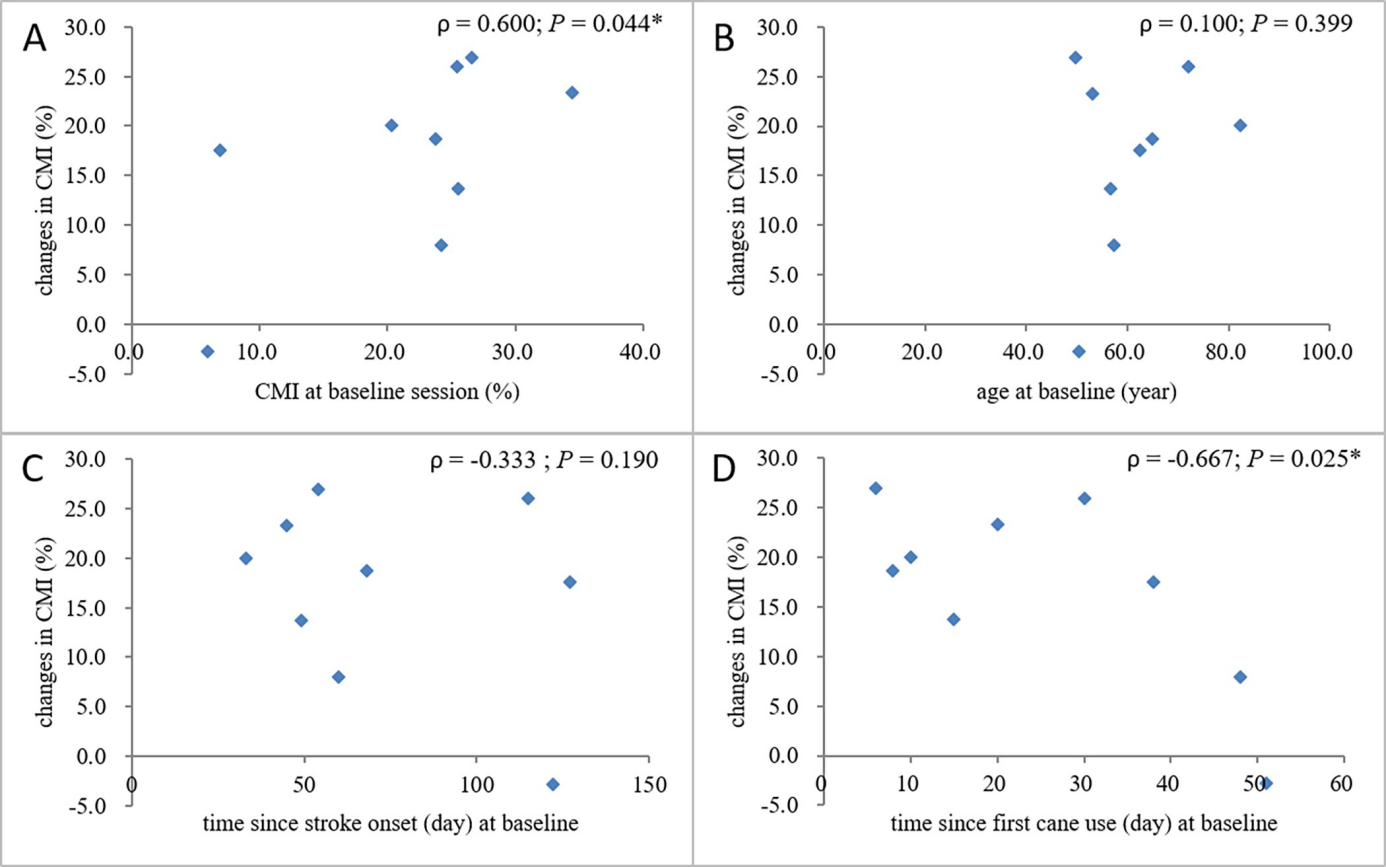
Scatter plots showing the relationship between the change in walking velocity CMI between the baseline and final sessions and (A) CMI at the baseline session, (B) age, (C) time since stroke onset, and (D) time since first cane use. A positive CMI value indicated deterioration due to dual-tasking. The results of the Spearman correlation tests are shown.

**Fig 4:**
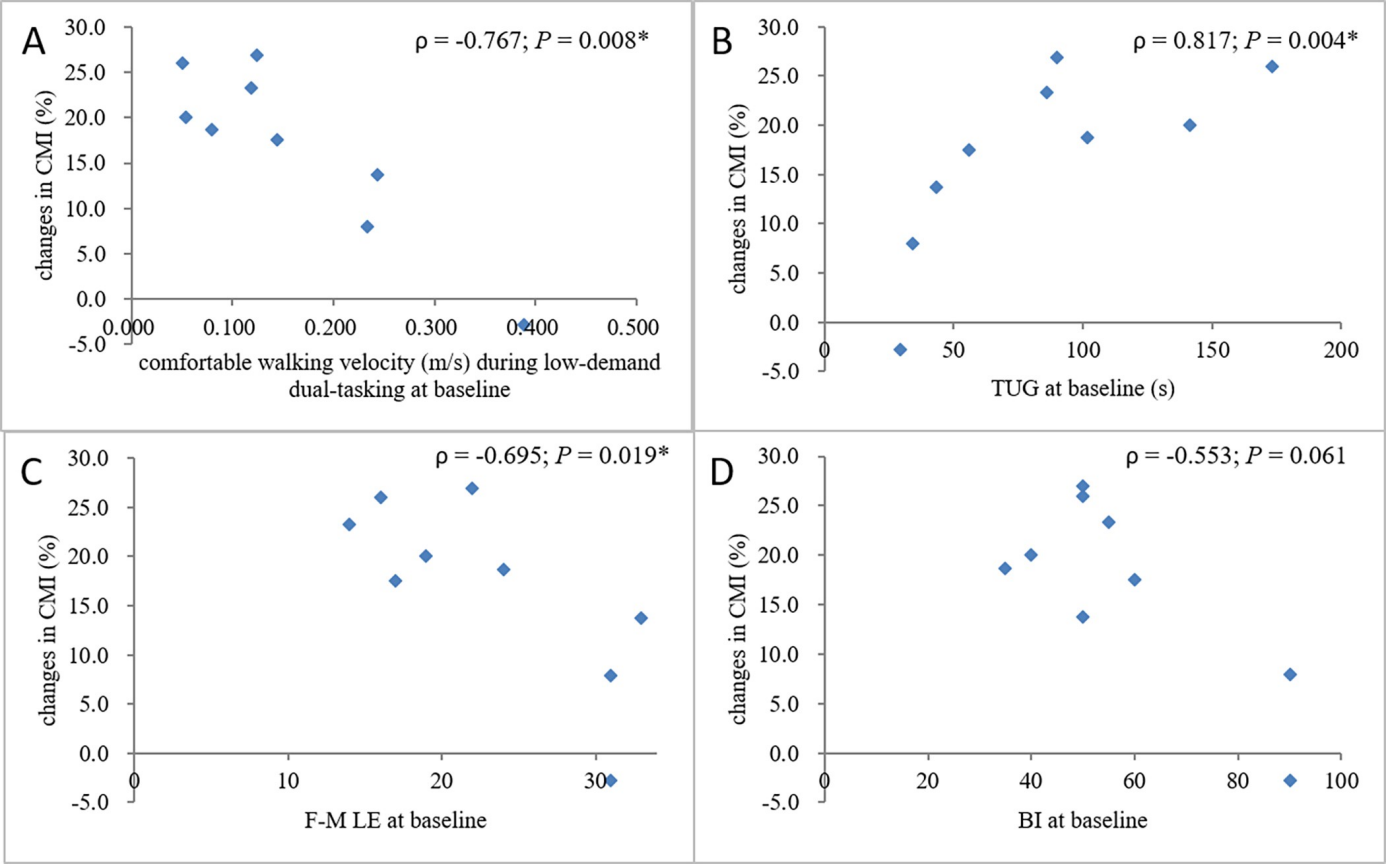
Scatter plots showing the relationship between the change in walking velocity CMI between the baseline and final sessions and (A) comfortable walking velocity during low-demand dual-tasking, (B) the Timed Up and Go test, (C) the lower extremity part of the Fugl-Meyer Assessment, and (D) the Barthel index. A positive CMI value indicated deterioration due to dual-tasking. The results of the Spearman correlation tests are shown.

Figs 3 and 4 reveal the relationship between changes in the extent of CMI of the walking velocity and other factors. The Spearman correlation tests revealed a moderate-to-good negative coefficient of time since first cane use (ρ = -0.667; p = 0.025), suggesting that the sooner the physical therapist confirmed that the participant had an independent walking ability with a quad cane after one had been prescribed for him/her, the greater the later improvement in CMI.

There was a moderate-to-good positive coefficient of baseline CMI (ρ = 0.600; p = 0.044), F-M LE (ρ = -0.695; p = 0.019), good-to-excellent negative coefficient of comfortable walking velocity (ρ = -0.767; p = 0.008), and good-to-excellent positive coefficient of TUG (ρ = 0.817; p = 0.004), suggesting that the worse the baseline performance of dual-tasking, motor control ability, walking velocity, and functional mobility, the greater the later improvement in CMI.

## Discussion

### Changes in the extent and pattern of CMI over time

In this pilot study, all 9 participants showed deteriorated dual-tasking performance in walking velocity at baseline. In contrast to our results, Cockburn et al. [6] excluded 30 out of 50 eligible patients with mixed neurological etiologies, because they did not show deteriorated dual-tasking performance. This discrepancy might be due to different chronicity post stroke: in our study with all subacute patients, decrement of gait performance due to dual-tasking was observed in all participants at baseline, whereas in Cockburn et al. [6] with patients of subacute and mostly chronic stages, many patients did not show gait decrement due to dual-tasking.

In our study, 8 out of the 9 participants showed a statistically significant improvement in CMI over time, suggesting that during the learning process of cane walking a certain level of automaticity is regained. In fact in 2 of our participants, the CMI values were negative, which means better performance due to dual-tasking. Enhanced postural control during dual-tasking has been reported in numerous studies, especially with easier tasks and in young adults [32], during which the focus of attention is shifted away from the postural task and the automaticity of postural task is assured. No previous cross-sectional research in patients with stroke has reported negative CMI in terms of group average. However, although not statistically significant, Lord et al. [10] documented faster average walking velocity during dual-tasking as compared to single-tasking. Furthermore in line with our study, Cockburn et al. [6] reported the exclusion of 4 out of 50 eligible participants due to negative or zero CMI at the second test, and 7 of the 10 included patients with stroke of subacute and mostly chronic stages showed CMI improvement over time. This means that, no matter the stage of stroke chronicity, improvement of CMI could be observed over time in majority of patients. In addition, there seemed to be a trend toward larger changes in earlier learning phases in the majority of our participants, which will require further elucidation. This results is reasonable according to the law of practice [24], i.e., we could expect greater improvement during the early learning phase.

The changes over time in the CMI pattern in this pilot study are novel findings. We categorized the CMI pattern into 6 possibilities. Our participants showed a consistent array of pattern changes over time, from mutual interference at baseline in all participants to cognitive-related motor interference in 1 participant and finally, a motor-priority tradeoff in 3 participants. None of the other three patterns were evident in this study. These results were consistent with the findings of Plummer et al. [14], which reported that patients with stroke are more likely to show mutual interference and cognitive-related motor interference.

Our observed changes over time in the CMI pattern supports the hypothesis of Plummer et al. [14] that the CMI pattern may evolve with stroke chronicity. All participants in the initial sessions showed the pattern of mutual interference, and some maintained this pattern until their final session. The pattern of mutual interference might represent the most severe CMI pattern, indicating that the processing demands of both tasks exceed the total attentional capacity [33], and the participants failed to find a strategy that preserved at least one task. As time progressed, one of our participants adopted a new pattern of cognitive-related motor interference. Plummer et al. [14] speculated that, in order to maintain postural stability, participants might consciously slow down their gait and/or prioritize the cognitive task during dual-tasking, thus, creating the CMI pattern of cognitive-related motor interference. Interestingly, the participant showing cognitive-related motor interference, as well as two other participants, eventually adopted another new pattern of motor-priority tradeoff in their final session, which has not been previously reported in literature with cross-sectional design. These patients had been repeatedly tested using the dual-tasking paradigm, so that they might allocate more of their attentional resources to the gait task. As time progressed, probably after 2 to almost 4 years, patients with stroke may show similar CMI levels compared to healthy controls [4], and the pattern of no interference may appear [10,13].

### Relationship between changes in extent of CMI and other factors

This pilot study is the first to examine the factors associated with changes in the extent of CMI. Our results showed that greater improvement in CMI over time was moderately to strongly associated with worse baseline performance in dual-tasking, walking velocity, functional mobility, and motor control ability. At first glance, our results seem to contradict with previous cross-sectional studies that documented a greater CMI in gait was associated with a greater lower extremity motor impairment [34], slower walking velocity [5,34], and worsened ability to perform ADL [5]. However, the CMI in cross-sectional studies and changes of CMI in our study surely have different meanings. Considering all these factors together, worsened performance in motor control, walking velocity, or physical function is associated with a worse CMI performance but is associated with a greater potential for CMI improvement over time.

### Feasibility of the dual-tasking paradigm of cane walking in patients with subacute stroke & limitations

We experienced challenges in conducting the dual-tasking paradigm among the stroke population who had just been able to walk without personal help. First, only patients who could solve simple mathematical tasks could be recruited in this study. Pumpho et al. [27] reported that among 50 eligible patients with stroke, 26 had a subtraction problem. Among 13 cognitive tasks, the authors concluded that for patients who had subtraction problems, alphabet fluency is suitable for use in a dual-tasking paradigm, whereas for patients who can perform subtraction, continuous subtraction by 3s is recommended.

Second, our instructions to prioritize both tasks might not clearly explained to the participants, leading to better cognitive performance during cane walking as compared to sitting in over one-third of the sessions. Because these CMI patterns rarely occurred in the literature according to a review article [14], they were considered as participants not complying with our instructions to prioritize both tasks. However, after eliminating these CMI patterns from further analyses, both quantitative and qualitative data revealed promising results. As one previous study has found task prioritization effects of focus of instructions during dual-tasking [35], when conducting dual-tasking paradigm, instructions have to be clearly explained.

Third, the sample size was small. The conduction of this study was challenged by the frailty status of subacute patients who could walk without personal help but needed contact guard or supervision. The recruitment of participants was also challenged by the research design that required up to six sessions in 3 months. The small sample size limited our analyses to non-parametric statistics. However, the sample size was calculated with alpha set at 0.05 and power set at 0.95 [19], and the pilot results showed that the dual-tasking paradigm of cane walking is feasible in majority of patients with subacute stroke.

Fourth, some patients with dragging gait and overlapped strides caused difficulty with the GAITRite analysis, forcing us to use stopwatch data in 6 out of 30 sessions. However, the data acquired using a manual stopwatch were equally satisfactory (the correlation between data obtained using GAITRite and the stopwatch was excellent; ρ = 0.995; *P* < 0.001). Fifth, the extension of walking surface by yoga mats might create changes of gait. Surface of yoga mat is slightly softer than the surface of GAITRite mat, minimizing the effects of acceleration and deceleration. However, the phases of acceleration and deceleration were not analyzed, and our patients of stroke walked at an extremely slow pace. Thus, this limitation would not seriously influence our results.

Sixth, the judgement of independent walking was subjective among different physical therapists. The baseline session was tested as soon as the physical therapist confirmed that the participant was able to walk independently, and this period varied widely in terms of the time after stroke onset (33–127 days) and the time after first cane use (6–51 days). This was probably due to the different type of intervention that influenced the judgement: the Bobath approach [36] or the motor relearning approach [37]. It is highly possible that therapists relying on the Bobath [36] approach opposed early walking with compensated patterns, therefore, prescribing canes for patients and confirming independent walking later than those relying on the motor relearning [37] approach who encouraged early walking, even with a suspended harness. As a result, although comfortable walking velocity and functional mobility at baseline revealed severe mobility problems in our participants, stroke severity, motor control ability, and function of daily living activities varied from mild to severe. Our correlation analyses showed that the sooner the physical therapist confirmed that the participant had an independent walking ability with a quad cane after prescription (i.e., the gait pattern was deemed to be acceptable), the greater the later improvement in CMI. Therefore, for efficient and safe cane walking during dual-tasking, the philosophy of early walking and task-oriented practices seems valid.

## Conclusions

In this pioneer work in stroke patients who had just learnt to walk with a cane in the subacute stage, conducting a dual-tasking paradigm was challenging. The instructions about task prioritization have to be clearly explained. However, even with just two trials each in low-demand and high-demand cane walking in participants who could perform subtractions, the dual-tasking paradigm could reveal CMI in all participants at the initial test and a subsequent improvement in CMI over time in most participants. In summary, the dual-tasking paradigm is feasible in patients with subacute stroke. A certain level of automaticity is regained during rehabilitation with cane walking, with larger improvements in more severely affected patients and a possibly faster recovery in earlier learning phases. There seems to be a certain path of evolution of CMI patterns over time: mutual interference, cognitive-related motor interference, and finally, motor-priority tradeoff. The CMI pattern provides information besides the extent of CMI on the attentional demand of cane walking and the strategies adopted by patients. Future studies should further explore these issues with larger sample sizes.

## Supporting information

S1 File (XLSX)

## References

[1] Lacour M, Bernard-Demanze L, Dumitrescu M. Posture control, aging, and attention resources: models and posture-analysis methods. Neurophysiol Clin. 2008;38(6):411–21. doi: 10.1016/j.neucli.2008.09.005 19026961

[2] Woollacott M, Shumway-Cook A. Attention and the control of posture and gait: a review of an emerging area of research. Gait Posture. 2002;16(1):1–14. doi: 10.1016/s0966-6362(01)00156-4 12127181

[3] Pashler H. Dual-task interference in simple tasks: data and theory. Psychol Bull. 1994;116(2):220–44. doi: 10.1037/0033-2909.116.2.220 7972591

[4] Canning CG, Ada L, Paul SS. Is automaticity of walking regained after stroke? Disabil Rehabil. 2006;28(2):97–102. doi: 10.1080/09638280500167712 16393839

[5] Haggard P, Cockburn J, Cock J, Fordham C, Wade D. Interference between gait and cognitive tasks in a rehabilitating neurological population. J NeurolNeurosurg Psychiatry. 2000;69(4):479–86. 10.1136/jnnp.69.4.479 PMC1737140 10990508

[6] Cockburn J, Haggard P, Cock J, Fordham C. Changing patterns of cognitive-motor interference (CMI) over time during recovery from stroke. Clin Rehabil. 2003;17(2):167–73. doi: 10.1191/0269215503cr597oa 12625657

[7] Plummer-D’Amato P, Altmann LJ, Behrman AL, Marsiske M. Interference between cognition, double-limb support, and swing during gait in community-dwelling individuals poststroke. Neurorehabil Neural Repair. 2010;24(6):542–9. doi: 10.1177/1545968309357926 20424190 PMC2923473

[8] Plummer-D’Amato P, Altmann LJ, Saracino D, Fox E, Behrman AL, Marsiske M. Interactions between cognitive tasks and gait after stroke: a dual task study. Gait Posture. 2008;27(4):683–8. doi: 10.1016/j.gaitpost.2007.09.001 17945497 PMC2913384

[9] Regnaux JP, David D, Daniel O, Smail DB, Combeaud M, Bussel B. Evidence for cognitive processes involved in the control of steady state of walking in healthy subjects and after cerebral damage. Neurorehabil Neural Repair. 2005;19(2):125–32. doi: 10.1177/1545968305275612 15883356

[10] Lord SE, Rochester L, Weatherall M, McPherson KM, McNaughton HK. The effect of environment and task on gait parameters after stroke: A randomized comparison of measurement conditions. Arch Phys Med Rehabil. 2006;87(7):967–73. doi: 10.1016/j.apmr.2006.03.003 16813785

[11] Kizony R, Levin MF, Hughey L, Perez C, Fung J. Cognitive load and dual-task performance during locomotion poststroke: a feasibility study using a functional virtual environment. Phys Ther. 2010;90(2):252–60. doi: 10.2522/ptj.20090061 20023003

[12] Kemper S, McDowd J, Pohl P, Herman R, Jackson S. Revealing language deficits following stroke: the cost of doing two things at once. Neuropsychol Dev Cogn B Aging Neuropsychol Cogn. 2006;13(1):115–39. doi: 10.1080/13825580500501496 16766346

[13] Dennis A, Dawes H, Elsworth C, Collett J, Howells K, Wade DT, et al. Fast walking under cognitive-motor interference conditions in chronic stroke. Brain Res. 2009;1287:104–10. doi: 10.1016/j.brainres.2009.06.023 19527695

[14] Plummer P, Eskes G, Wallace S, Giuffrida C, Fraas M, Campbell G, et al. Cognitive-motor interference during functional mobility after stroke: state of the science and implications for future research. Arch Phys Med Rehabil. 2013;94(12):2565–74 e6. doi: 10.1016/j.apmr.2013.08.002 23973751 PMC3842379

[15] Lucki K, Bach M, Banzer W, Vogt L. Walker use affects Timed Up and Go and gait speed measures. J Am Geriatr Soc. 2009;57(10):1963–5. doi: 10.1111/j.1532-5415.2009.02475.x 19807815

[16] Wright DL, Kemp TL. The dual-task methodology and assessing the attentional demands of ambulation with walking devices. Phys Ther. 1992;72(4):306–12. doi: 10.1093/ptj/72.4.306 1584862

[17] Wellmon R, Pezzillo K, Eichhorn G, Lockhart W, Morris J. Changes in dual-task voice reaction time among elders who use assistive devices. J Geriatr Phys Ther. 2006;29(2):74–80. doi: 10.1519/00139143-200608000-00006 16914064

[18] Chen HY, Chen HI, Fu SY, Hesieh YW. Attentional demands of cane-free walking and cane walking in subacute stroke patients who have just learned to walk without a cane. Int J Rehabil Res. 2021;44(4):377–81. doi: 10.1097/MRR.0000000000000488 34380994

[19] Faul F, Erdfelder E, Buchner A, Lang AG. Statistical power analyses using G*Power 3.1: Tests for correlation and regression analyses. Behav Res Methods 2009;41:1149–60. doi: 10.3758/BRM.41.4.1149 19897823

[20] Borson S, Scanlan J, Brush M, Vitaliano P, Dokmak A. The mini-cog: a cognitive ’vital signs’ measure for dementia screening in multi-lingual elderly. Int J Geriatr Psychiatry. 2000;15(11):1021–7. doi: 10.1002/1099-1166(200011)15:11&lt;1021::aid-gps234&gt;3.0.co;2-6 11113982

[21] Podsiadlo D, Richardson S. The timed "Up & Go": a test of basic functional mobility for frail elderly persons. J Am Geriatr Soc. 1991;39(2):142–8. 1991946 10.1111/j.1532-5415.1991.tb01616.x

[22] Fugl-Meyer AR, Jaasko L, Leyman I, Olsson S, Steglind S. The post-stroke hemiplegic patient. 1. a method for evaluation of physical performance. Scand J Rehabil Med. 1975;7(1):13–31. 1135616

[23] Mahoney FI, Barthel DW. Functional evaluation: The Barthel Index. Md State Med J. 1965;14:61–5. 14258950

[24] Schmidt RA, Lee TD. Motro Control and Learning: A Behavioral Emphasis. Champaign, IL: Human Kinetics; 1999.

[25] Chen HY, Tang PF. Factors contributing to single- and dual-task Timed "Up & Go" test performance in middle-aged and older adults who are active and dwell in the community. Phys Ther. 2016;96(3):284–92. 26183585 10.2522/ptj.20140292

[26] Al-Yahya E, Dawes H, Smith L, Dennis A, Howells K, Cockburn J. Cognitive motor interference while walking: a systematic review and meta-analysis. Neurosci Biobehav Rev. 2011;35(3):715–28. doi: 10.1016/j.neubiorev.2010.08.008 20833198

[27] Pumpho A, Chaikeeree N, Saengsirisuwan V, Boonsinsukh R. Selection of the better dual-Timed Up and Go cognitive task to be used in patients with stroke characterized by subtraction operation difficulties. Front Neurol. 2020;11:262. doi: 10.3389/fneur.2020.00262 32390925 PMC7190870

[28] Chu YH, Tang PF, Peng YC, Chen HY. Meta-analysis of type and complexity of a secondary task during walking on the prediction of elderly falls. Geriatr Gerontol Int. 10.1111/j.1447-0594.2012.00893.x 22694365

[29] Fraizer EV, Mitra S. Methodological and interpretive issues in posture-cognition dual-tasking in upright stance. Gait Posture. 2008;27(2):271–9. doi: 10.1016/j.gaitpost.2007.04.002 17524648

[30] McDowd JM. The effects of age and extended practice on divided attention performance. J Gerontol. 1986;41(6):764–9. doi: 10.1093/geronj/41.6.764 3772053

[31] Portney LG, Watkins MP, editors. Foundations of Clinical Research: Applications to Practice. 2nd ed. Upper Saddle River: NJ: Prentice Hall; 2000.

[32] Huxhold O, Li SC, Schmiedek F, Lindenberger U. Dual-tasking postural control: aging and the effects of cognitive demand in conjunction with focus of attention. Brain Res Bull. 2006;69(3):294–305. doi: 10.1016/j.brainresbull.2006.01.002 16564425

[33] Kahneman D. Attention and Effort. Englewood Cliffs, NJ: Prentice Hall; 1973.

[34] Plummer-D’Amato P, Altmann LJ. Relationships between motor function and gait-related dual-task interference after stroke: a pilot study. Gait Posture. 2012;35(1):170–2. doi: 10.1016/j.gaitpost.2011.08.015 21962406

[35] Verghese J, Kuslansky G, Holtzer R, Katz M, Xue X, Buschke H, et al. Walking while talking: effect of task prioritization in the elderly. Arch Phys Med Rehabil. 2007;88(1):50–3. doi: 10.1016/j.apmr.2006.10.007 17207675 PMC1894901

[36] Davis P. Steps to Follow. 2nd ed. New York: Springer; 2000.

[37] Carr JS R. Stroke Rehabilitation: Guidelines for Exercise and Training to Optimize Motor Skill. First ed. London: Butterworth-Heinemann; 2003.

